# Varianz der operativen Versorgung bei dislozierten Schenkelhalsfrakturen in zertifizierten AltersTraumaZentren DGU® und Endoprothetikzentren in Deutschland

**DOI:** 10.1007/s00113-025-01619-1

**Published:** 2025-08-25

**Authors:** Yasmin Hartmann, Katherine Rascher, Miguel Pishnamaz, Filippo Migliorini, Klemens Horst, Matthias Knobe, Frank Hildebrand, Christian David Weber, AltersTraumaRegister DGU

**Affiliations:** 1 München Klinik gGmbH, München, Deutschland; 2 AUC-Academy for Trauma Surgery (AUC), München, Deutschland; 3 Department of Orthopaedics, Trauma and Reconstructive Surgery, Klinik für Orthopädie, Unfall- und Wiederherstellungschirurgie, RWTH Aachen University Hospital, Pauwelsstraße 30, 52074 Aachen, Deutschland; 4 Department of Orthopaedic and Trauma Surgery, SABES Academic Hospital of Bolzano, Bolzano, Italien; 5 Klinik für Unfall- und orthopädische Chirurgie, St. Marien-Krankenhaus, Wüllener Str. 101, 48683 Ahaus, Deutschland; 6 Working Committee on Geriatric Trauma Registry (AK ATR) of the German Trauma Society (DGU), Berlin, Deutschland

**Keywords:** Instabile Schenkelhalsfraktur, Alterstraumatologie, AltersTraumaZentrum DGU®, Endoprothetikzentrum, Zertifizierung, Versorgungsvarianz, Unstable femoral neck fracture, Geriatric trauma, Geriatric Trauma Center, Arthroplasty center, Certification, Treatment variability

## Abstract

**Hintergrund:**

In Deutschland existiert eine spezielle Zertifizierungsstruktur, in der Kliniken als TraumaZentrum DGU®, AltersTraumaZentrum DGU® bzw. Endoprothetikzentrum (*endoCert*) zertifiziert werden können. Geriatrische Patienten mit dislozierten Schenkelhalsfrakturen stellen eine hochrelevante Entität dar. Die Struktur- und Prozessqualität wird im Rahmen einer Zertifizierung als AltersTraumaZentrum DGU® (*ATZ*) bzw. Endoprothetikzentrum (*EPZ*) validiert, dies stellt einen methodisch spannenden Ansatz zur Analyse der Versorgungsrealität dar.

**Fragestellung:**

Existiert zwischen zertifizierten *ATZ* und dual zertifizierten *ATZ* *+* *EPZ* eine Varianz der Versorgung im Hinblick auf gelenkerhaltende bzw. -ersetzende Operationsverfahren bei geriatrischen dislozierten Schenkelhalsfrakturen sowie Kurzzeitkomplikationen?

**Material und Methoden:**

Daten des AltersTraumaRegister DGU® (*ATR-DGU*) wurden aus 46 Kliniken mit *ATZ* und 52 Kliniken mit *ATZ* *+* *EPZ* analysiert. Der Beobachtungszeitraum umfasste den stationären Klinikaufenthalt und ein 120-tägiges Nachbeobachtungsintervall. Der primäre Endpunkt war die Mortalität, sekundäre Endpunkte umfassten die Mobilität, Reoperationen und den Gesundheitsstatus. Es erfolgten univariate und multivariate Analysen zur Identifikation von Odds ratios (OR) nach Adjustierung für Alter, Geschlecht, ASA-Score und Begleitverletzungen.

**Ergebnisse:**

Das mediane Alter des Kollektivs (*n* = 7389) betrug 84 Jahre, 29,6 % bzw. 29,8 % waren männlich, die mediane Dauer bis zur Operation betrug 20,9 h (*ATZ*) bzw. 20,5 h (*ATZ* *+* *EPZ*), und die mediane Liegedauer betrug in beiden Versorgungsstrukturen 15,1 Tage. Die Zahl gelenkerhaltender Eingriffe war in *ATZ* signifikant gegenüber Kliniken mit dualer Zertifizierung erhöht (*ATZ*: 8,6 % vs. *ATZ* *+* *EPZ*: 2,6 %; OR = 3,63). Die Reoperationsrate war im Primäraufenthalt vergleichbar (3,7 % vs. 3,9 %), im 120-Tages Verlauf jedoch signifikant in *ATZ* *+* *EPZ*-Kliniken erhöht (4,1 % vs. 6,0 %; *p* = 0,022). Revisionen aufgrund von periprothetischen Frakturen erfolgten signifikant häufiger in *ATZ *ohne *EPZ *(8,2 % vs. 3,5 %). In der multivariaten Analyse zeigte sich für Kliniken mit dualer Zertifizierung in der Akutphase eine erhöhte Mortalität (OR 1,26; 1,02–1,56; *p* = 0,031), im 120-Tages Verlauf eine erhöhte Rate an Reoperationen (OR 1,45; 1,06–2,02; *p* = 0,024) und stationären Wiederaufnahmen (OR 1,42, 1,02–2,00; *p* = 0,043).

**Schlussfolgerung:**

In Deutschland besteht bei geriatrischen dislozierten Schenkelhalsfrakturen eine institutionelle Versorgungsvarianz. In alterstraumatologisch zertifizierten Kliniken ohne Endoprothetikzentrum zeigt sich eine signifikant erhöhte Rate an gelenkerhaltenden Versorgungen mit Unterschieden in Bezug auf die Morbidität und Mortalität in der Akutphase.

**Graphic abstract:**

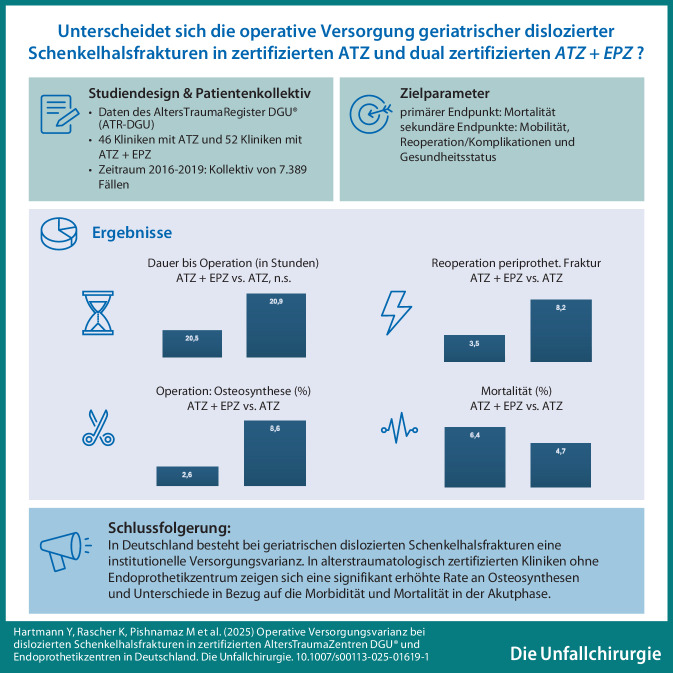

## Abstract (en)

**Background:**

There is an almost unique certification structure in Germany in which clinics can be certified either as trauma center DGU® (TraumaZentrum DGU®), geriatric trauma center DGU® (AltrsTraumaZentrum DGU®) or arthroplasty center (endoCert), among others. Geriatric patients with displaced femoral neck fractures represent a highly relevant entity. The structural and process quality are validated within the framework of certification as both an AltersTraumaZentrum DGU® (ATZ) and arthroplasty center (EPZ), which represents a methodologically challenging approach to the analysis of treatment reality.

**Objective:**

Is there a treatment variation between certified geriatric trauma centers (ATZ) and dual certified geriatric and arthroplasty centers (ATZ+EPZ) with respect to joint-preserving or joint replacement surgical procedures for geriatric displaced femoral neck fractures and short-term complications?

**Material and methods:**

Data from the Registry for Geriatric Trauma of the German Trauma Society (ATR-DGU) from 46 clinics with ATZ and 52 clinics with ATZ + EPZ were analyzed. The follow-up period included both the in-hospital stay and a 120-day follow-up interval. The primary outcome was mortality, secondary endpoints included mobility, reoperations and health status. Univariate and multivariate analyses were performed to calculate odds ratios (OR) after adjustment for age, gender, ASA score and concomitant injuries.

**Results:**

The median age of the collective (*n* = 7389) was 84 years, 29.6% and 29.8% respectively were male, the median time until surgery was 20.9 h (ATZ) vs. 20.5 h (ATZ + EPZ) and the median length of stay was 15.1 days for both types of center. The number of joint-preserving interventions was significantly increased in ATZ compared to clinics with dual certification (ATZ: 8.6% vs. ATZ + EPZ: 2.6%; OR = 3.63). The reoperation rate was comparable in the primary stay (3.7% vs. 3.9%) but was significantly increased over the 120-day course in clinics with dual certification (4.1% vs. 6.0%; *p* = 0.022). Revisions due to periprosthetic fractures occurred more frequently in ATZ without EPZ (8.2% vs. 3.5%). The multivariate analysis showed an increased mortality in the acute phase (OR 1.26; 1.02–1.56; *p* = 0.031), an increased rate of reoperations in the 120-day course (OR 1.45; 1.06–2.02; *p* = 0.024) and inpatient readmissions (OR 1.42, 1.02–2.00; *p* = 0.043) for centers with dual certification.

**Conclusion:**

In Germany an institutional treatment variation for geriatric displaced femoral neck fractures exists. In certified geriatric trauma centers without certified arthroplasty center there is a significantly increased rate of joint-preserving treatment with differences in terms of morbidity and mortality in the acute phase.

## Einführung

Geriatrische Traumapatienten stellen ein besonderes Patientenkollektiv, welches evidenzbasierte, interdisziplinäre als auch individualisierte Behandlungsstrategien erfordert, dar [1]. Instabile Schenkelhalsfrakturen gehören zu den relevantesten Verletzungen des Menschen, da sie mit massiven individuellen und gesellschaftlichen Auswirkungen verbunden sind [2]. Die meisten Patienten erlangen nach einer Fraktur des femoralen Schenkelhalses ihre Lebensqualität und Mobilität nicht vollumfänglich wieder [3]. Viele aktivere ältere Patienten verlieren ihre eigenständige Mobilität, gebrechlichere Patienten ihr unabhängiges Leben zu Hause, und die gebrechlichsten werden durch Schmerzen, weitere Mobilitätseinbuße und Unfähigkeit zur Selbstversorgung zusätzlich geschwächt [4, 5].

Für dislozierte Schenkelhalsfrakturen sind sowohl gelenkerhaltende als auch gelenkersetzende Versorgungtechniken etabliert [6]. In der aktuellen Literatur existieren signifikante Versorgungsunterschiede in der Behandlung von dislozierten Schenkelhalsfrakturen. Die Form der operativen Versorgung wird neben patientenabhängigen Faktoren auch von strukturellen Faktoren beeinflusst.

Die Zertifizierung von Zentren zielt darauf ab, die Behandlung zu standardisieren, um die Versorgungsqualität zu optimieren und die Patientensicherheit zu verbessern [7]. Mit der Einführung der Qualitätsinitiative „Endoprothesenregister Deutschland (EPRD)“ und der Zertifizierung von Endoprothetikzentren seit 2012 soll die Endoprothetik in Deutschland besser dokumentiert sowie qualitätsgesichert durchgeführt und weiterentwickelt werden [8]. Seit 2014 können sich in Deutschland Krankenhäuser, in denen Patienten interdisziplinär unfallchirurgisch-geriatrisch behandelt werden, als AltersTraumaZentrum DGU® zertifizieren. Für die Messung der Behandlungsqualität in diesen Zentren wurde das AltersTraumaRegister DGU® aufgebaut [9]. Eine aktuelle Analyse von Traumazentren mit teils dualer Zertifizierung als Alterstraumazentrum konnte keinen Überlebensvorteil bei schwer verletzten geriatrischen Patienten identifizieren [10]. Die vorliegende Studie zielt darauf ab, die aktuelle Versorgungsrealität zu analysieren, die Bedeutung der verschiedenen Einflussfaktoren zu quantifizieren und infrastrukturelle Unterschiede zwischen zertifizierten AltersTraumaZentren DGU® mit bzw. ohne additive Zertifizierung als Endoprothetikzentrum und deren Einfluss auf den Zeitpunkt und die Rate des gelenkerhaltenden bzw. -ersetzenden Vorgehens zu beurteilen.

## Material und Methoden

### Studiendesign und Patientenauswahl

Wir führten eine retrospektive, registerbasierte Beobachtungsstudie durch mit Datensätzen aus dem AltersTraumaRegister DGU® (ATR-DGU), die geriatrische Patienten mit medialen Schenkelhalsfrakturen (*n* = 10.795) aus 98 Kliniken mit AltersTraumaZentrum DGU® und Endoprothetikzentrum im Zeitraum zwischen 01.01.2016 und 31.12.2019 einschlossen. Innerhalb des Kollektivs von 98 Kliniken mit einem zertifizierten AltersTraumaZentrum DGU® (*ATZ-Gruppe*) konnten über die Datenbank (EndoMap, Service der Fa. Clarcert) insgesamt 52 Kliniken identifiziert werden, die gleichzeitig als Endoprothetikzentrum zertifiziert waren (*ATZ* *+* *EPZ-Gruppe*).

**Abb. 1:**
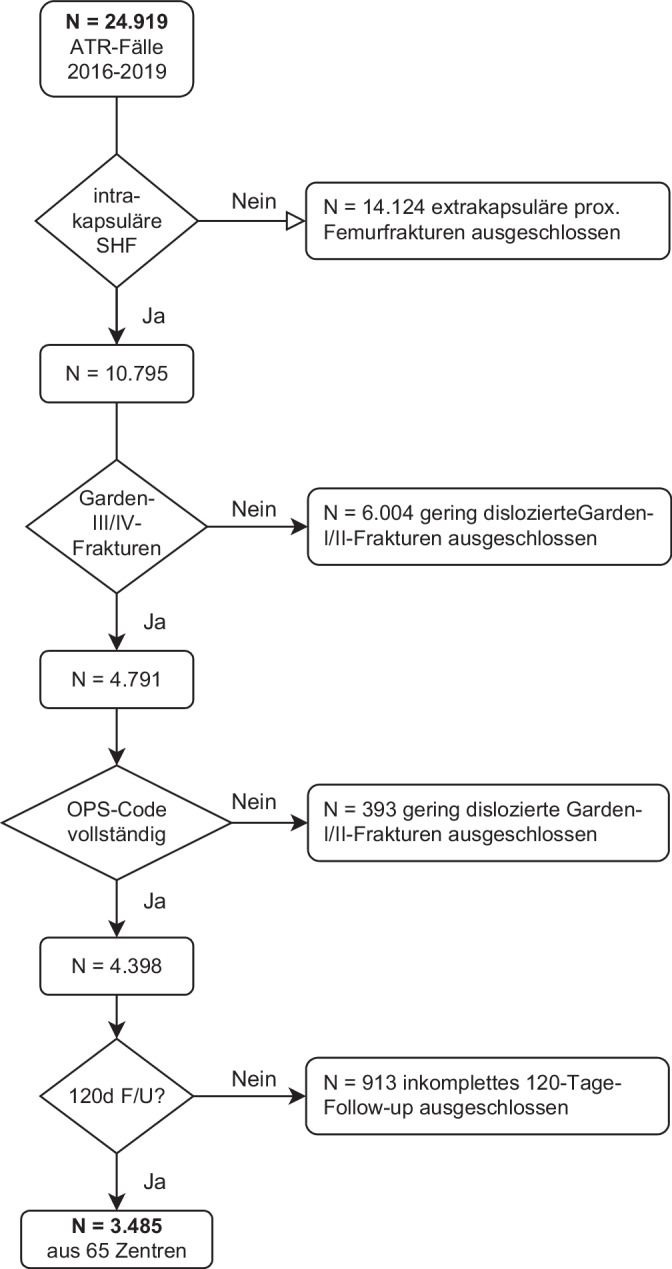
Flussdiagramm Patientenrekrutierung; *ATR* AltersTraumaRegister DGU® (ATR-DGU), *SHF* Schenkelhalsfraktur, *OPS* Operationen- und Prozedurenschlüssel, *F/U* Follow-up

Nach Ausschluss der undislozierten Frakturen verblieb eine Patientenkohorte mit dislozierten Schenkelhalsfrakturen (Garden-III/IV-Fraktur) (*n* = 7491) und Angaben zur erfolgten operativen Versorgung (*n* = 7389) mittels Endoprothese (*n* = 6999) oder gelenkerhaltender Osteosynthese (*n* = 390) zur Auswertung (Abb. 1).

Die Daten des ATR-DGU umfassen eine pseudonymisierte und standardisierte Dokumentation geriatrischer Patienten (≥ 70 Jahre) mit operativ behandelten proximalen Femurfrakturen, die zu 5 Zeitpunkten der Behandlung erhoben wurden: bei der stationären Aufnahme ins Krankenhaus, präoperativ, intraoperativ, 7 Tage postoperativ und bei Entlassung. Darüber hinaus wurde am 120. postoperativen Tag eine zusätzliches optionales Follow-up (*n* = 3485) durchgeführt.

Bei der Aufnahme ins Krankenhaus wurde ein Screening zur Erstidentifikation der geriatrischen Patienten „Identification of Seniors at Risk“ (ISAR) durchgeführt [7, 8]. Der ISAR-Score gibt das Risiko negativer gesundheitlicher Folgen an, einschließlich Mortalität, Funktionseinbußen, Rückübernahme und Heimeinweisung, und reicht von 0 (geringes Risiko) bis 6 (hohes Risiko) Punkten. Darüber hinaus wurden die Anzahl der Patienten, die präoperativ Antikoagulanzien eingenommen hatten, erfasst sowie die genaue Medikation und der letzte präoperative Einnahmezeitpunkt. Alle Patienten gaben nach Erhalt mündlicher und schriftlicher Informationen eine schriftliche Einverständniserklärung zur Teilnahme am AltersTraumaRegister DGU® (ATR-DGU) ab.

Die vorliegende Studie entspricht der RECORD-Erklärung [11] und steht im Einklang mit den Publikationsrichtlinien des ATR-DGU [12], registriert unter der Projekt-ID 2019–009.

## Primäre und sekundäre Ergebnisse

Der primäre Endpunkt war die Sterblichkeit sowohl während der stationären Behandlung als auch 120 Tage nach der Operation. Zu den sekundären Endpunkten der Auswertung gehören die Liegedauer im Krankenhaus, das Vorkommen von zusätzlichen behandlungsbedürftigen Verletzungen, die Versorgungsart (gelenkerhaltend vs. Gelenkersatz), die Zeit zwischen stationärer Aufnahme und operativer Versorgung, die Reoperationsrate und die 7‑Tage-Mobilität der Patienten. Die Gehfähigkeit vor dem Frakturereignis wird unterteilt in selbstständige Gehfähigkeit ohne Hilfsmittel und Gehfähigkeit mit Hilfsmitteln oder keine funktionelle Gehfähigkeit. Bei Gehfähigkeit mit Hilfsmitteln wurde zusätzlich die Art des Hilfsmittels (Gehstock/Gehstütze oder 2 Gehstützen/Rollator) und die Mobilität außer oder nur im Haus erfasst. Darüber hinaus wurde erfasst, wohin der Patient aus der stationären Behandlung entlassen wurde, z. B. nach Hause bzw. in betreutes Wohnen, Heim oder stationäre Einrichtung.

Als Reoperation während des Aufenthalts wurde jede chirurgische Maßnahme klassifiziert, die infolge von Komplikationen der gleichen koxalen Femurfraktur durchgeführt wurde. *Dies schließt Repositionen nach Luxation ein, jedoch ohne eine Differenzierung zwischen geschlossener und offener Reposition vorzunehmen.*

## Statistische Auswertung

Die Basismerkmale und Ergebnisvariablen der Studienpopulation werden mittels deskriptiver Statistiken bereitgestellt. Die Daten werden als Mediane mit Interquartilsbereichen (IQR) für kontinuierliche Variablen oder als Prozentsätze (%) für kategorische Variablen dargestellt.

Der primäre Endpunkt war die Mortalität; sekundäre Endpunkte umfassten die Mobilität, Reoperationen und den Gesundheitsstatus. Es erfolgten univariate und multivariate Analysen zur Identifikation von Odds ratios (OR) nach Adjustierung für Alter, Geschlecht, ASA-Score und Begleitverletzungen.

## Ergebnisse

Im Zeitraum von 2016 bis 2019 haben die teilnehmenden Zentren ein Kollektiv von 7389 Fällen mit dislozierter Schenkelhalsfraktur erfasst, hierunter befanden sich 2189 männliche Patienten (29,7 %) und 5184 Patientinnen (70,3 %) im medianen Alter von 84 Jahren (Tab. 1).

**Tab. 1:** Univariate Analyse – Gesamtkohorte (*n* = 7389 Patienten aus 98 Kliniken)

	Gesamtkohorte
**Anzahl, Kliniken**	98
**Anzahl, Patienten**	7389
**Alter (in Jahren)** – Median (IQR)	84 (79; 89)
**Geschlecht**	
Männlich	2189 (29,7 %)
Weiblich	5184 (70,3 %)
**ASA**	
Unbekannt	68
1	85 (1,2 %)
2	1571 (21,5 %)
3	5060 (69,2 %)
4	586 (8,0 %)
5	12 (0,2 %)
**ISAR**	
0	409 (8,4 %)
1	588 (12,1 %)
2	1004 (20,7 %)
3	1161 (24,0 %)
4	1059 (21,9 %)
5	485 (10,0 %)
6	138 (2,9 %)
**Zusätzliche Verletzung**	
Unbekannt	14
Nein	6747 (91,7 %)
Ja	608 (8,3 %)
**Reoperation während des Aufenthalts**	
Unbekannt	10
Nein	7096 (96,2 %)
Ja	283 (3,8 %)
**Reoperationstyp** *(Mehrere Operationsverfahren pro Patient möglich)*	
Reposition	36 (11,4 %)
Spülung/Débridement	141 (44,6 %)
Entfernung von Implantat	20 (6,3 %)
Girdlestone-Operation	5 (1,6 %)
Periprothetische Fraktur	18 (5,7 %)
Sonstiges	96 (30,4 %)
Versorgung/Operationstyp	
**Gelenkerhalt **(Schrauben, DHS, Marknagel, sonstige osteosynthetische Operationsverfahren)	390 (5,3 %)
**Endoprothetik **(Duokopf, Totalendoprothese)*	6999 (94,7 %)
**Prothesentyp**	
Duokopfprothese	5652 (80,8 %)
Totalendoprothese	1342 (19,2 %)
**„Time to surgery“ (h)** – Median (IQR)	20,7 (11,8, 29,9)
**Liegedauer im KH (Tage)** – Median (IQR)	15,1 (10,1, 22,0)
**Entlassen nach/in**	
Zu Hause/betreutes Wohnen	1656 (24,7 %)
Heim	1742 (26,0 %)
Stationäre Aufnahme	3295 (49,2 %)
**„7-Tage-Walking-Ability“**	
Unbekannt	175
Selbstständig	62 (0,9 %)
Unterarmstützen	917 (12,9 %)
Rollator	2315 (32,5 %)
Nicht möglich	1354 (19,0 %)
Gehbock	1096 (15,4 %)
Gehwagen	1387 (19,5 %)
**Mortalität im Krankenhaus**	
Lebend	6953 (94,4 %)
Tot	415 (5,6 %)
**120-Tage-Follow-up**	
**Patienten mit geschlossenem Follow-up**	*n* = 3485 (47 %)
**Mortalität (KH-Entlassung bis 120 Tage)**	
Lebend	2491 (87,6 %)
Tot	352 (12,4 %)
**Wiederaufnahme (KH-Entlassung bis 120 Tage)**	
Unbekannt	76
Nein	3147 (94,7 %)
Ja	177 (5,3 %)
**Reoperation (120-Tage-Follow-up)**	
Unbekannt	204
Nein	2981 (94,8 %)
Ja	163 (5,2 %)

Die operative Behandlung der Betroffenen erfolgte in 46 zertifizierten AltersTraumaZentren DGU® (ATZ-Gruppe: *n* = 3353; 45,4 %) bzw. an 52 Kliniken mit dualer Zertifizierung als ATZ und Endoprothetikzentrum (ATZ/EPZ-Gruppe: *n* = 4036; 54,6 %). Die demografischen Basischarakteristika wie das mediane Alter (84 Jahre, IQR 79; 89), die Geschlechtsverteilung und der ISAR-Score waren in beiden Hauptgruppen vergleichbar.

Im zeitlichen Verlauf der operativen bzw. stationären Behandlung zeigte sich kein signifikanter Unterschied zwischen Kliniken mit einfacher bzw. dualer Zertifizierung, das Zeitfenster bis zur Operation betrug in ATZ/EPZ-Kliniken 20,5 h und in ATZ-Kliniken 20,9 h (*p* = 0,068), die mediane stationäre Aufenthaltsdauer betrug für beide Gruppen 15,1 Tage (*p* *=* *0,507)* (Tab. 2).

**Tab. 2:** Univariate Analyse – *ATZ*-Kliniken vs. *ATZ* *+* *EPZ*-Kliniken

	*ATZ* *+* *EPZ*	*Nur ATZ*	*p*-Wert
**Anzahl, Kliniken**	52	46	–
**Anzahl, Patienten**	4036 (54,6 %)	3353 (45,4 %)	–
**Alter** (in Jahren) Median (IQR)	84 (79; 89)	84 (79; 89)	0,222*
**Geschlecht**			
Männlich	1190 (29,6 %)	999 (29,8 %)	0,854
Weiblich	2832 (70,4 %)	2352 (70,2 %)	
**ASA**			
Unbekannt	35	33	< 0,001
1	33 (0,8 %)	52 (1,6 %)	
2	791 (19,8 %)	780 (23,5 %)	
3	2793 (69,9 %)	2267 (68,3 %)	
4	376 (9,4 %)	210 (6,3 %)	
5	4 (0,1 %)	8 (0,2 %)	
**ISAR**			
0	239 (8,4 %)	170 (8,6 %)	0,113
1	343 (12,0 %)	245 (12,3 %)	
2	601 (21,0 %)	403 (20,3 %)	
3	708 (24,8 %)	453 (22,8 %)	
4	611 (21,4 %)	448 (22,5 %)	
5	289 (10,1 %)	196 (9,9 %)	
6	66 (2,3 %)	72 (3,6 %)	
**Zusätzliche Verletzung**			
Nein	3707 (92,3 %)	3040 (91,1 %)	0,067
Ja	310 (7,7 %)	247 (8,9 %)	
**Reoperation während des Aufenthalts**			
Nein	3869 (96,1 %)	3227 (96,3 %)	0,710
Ja	158 (3,9 %)	125 (3,7 %)	
**Reoperationstyp** (*Mehrere Operationsverfahren pro Patient möglich*)			
Reposition	18 (10,6 %)	18 (12,3 %)	–
Spülung/Débridement	74 (43,5 %)	67 (45,9 %)	
Entfernung von Implantat	13 (7,7 %)	7 (4,8 %)	
Girdlestone-Operation	5 (3,0 %)	0	
Periprothetische Fraktur	6 (3,5 %)	12 (8,2 %)	
Sonstiges	54 (31,8 %)	42 (28,8 %)	
**Versorgung/Operationstyp**			
*Gelenkerhalt* (Schrauben, DHS, Marknagel, sonstige osteosynthetische Operationsverfahren)	103 (2,6 %)	287 (8,6 %)	< 0,001
*Endoprothetik* (Duokopf, Total-Endoprothese)	3933 (97,4 %)	3066 (91,4 %)	
**Prothesentyp**			
Duokopfprothese	3111 (79,1 %)	2541 (83,0 %)	< 0,001
Totalendoprothese	822 (20,9 %)	520 (17,0 %)	
**„Time to surgery“** (h) – (*n*) *Median (IQR)*	(*n* = 4036) 20,5 (11,3, 29,5)	(*n* = 3353) 20,9 (12,5; 30,2)	0,068*
**Liegedauer im KH** (Tage) (*n*) *Median (IQR)*	(*n* = 4036) 15,1 (10,1, 21,0)	(*n* = 3353) 15,1 (10,0; 22,1)	0,507*
**Entlassen nach/in**			
Zu Hause/betreutes Wohnen	838 (23,3 %)	818 (26,4 %)	< 0,001
Heim	872 (24,3 %)	870 (28,0 %)	
Stationäre Einrichtung	1880 (52,4 %)	1415 (45,6 %)	
**„7-Tage-Gehfähigkeit“**			
Unbekannt	90	85	< 0,001
Selbstständig	27 (0,7 %)	35 (1,1 %)	
Unterarmstützen	480 (12,3 %)	437 (13,5 %)	
Rollator	1211 (31,1 %)	1104 (34,1 %)	
Nicht möglich	784 (20,1 %)	570 (17,6 %)	
Gehbock	507 (13,0 %)	589 (18,2 %)	
Gehwagen	887 (22,8 %)	500 (15,5 %)	
**Mortalität im Krankenhaus**			
Lebend	3770 (93,6 %)	3183 (95,3 %)	0,003
Tot	257 (6,4 %)	158 (4,7 %)	
**120-Tage-Follow up**			
**Patienten mit geschlossenem Follow-up**	2031 (50 %)	1454 (43,4 %)	–
**Mortalität (KH-Entlassung bis 120 Tage)**			
Lebend	1420 (88,0 %)	1071 (87,1 %)	0,475
Tot	193 (12,0 %)	159 (12,9 %)	
**Wiederaufnahme (KH-Entlassung bis 120 Tage)**			
Unbekannt	56	20	0,013
Nein	1807 (93,8 %)	1340 (95,9 %)	
Ja	119 (6,2 %)	58 (4,2 %)	
**Reoperation (120-Tage-Follow-up)**			
Unbekannt	126	78	0,022
Nein	1712 (94 %)	1269 (95,9 %)	
Ja	109 (6,0 %)	54 (4,1 %)	

*Mann-Whitney-U-Test, alle anderen Tests sind Chi-Squared Tests

*p* < 0,05

In Bezug auf die Form der operativen Therapie (Osteosynthese vs. Hemi‑/Endoprothese) zeigten sich signifikante Unterschiede zwischen den beiden Hauptgruppen, dislozierte Schenkelhalsfrakturen wurden an ATZ-Kliniken um ein Vielfaches häufiger gelenkerhaltend operiert (ATZ *n* = 287; 8,6 % vs. ATZ/EPZ *n* = 103; 2,6 %, *p* < 0,001). Auch in Bezug auf die endoprothetische Versorgung zeigten sich signifikante Unterschiede bei der Versorgung mit Hemi- oder Totalendoprothese. In ATZ/EPZ-Kliniken erfolgte eine signifikant häufigere Versorgung mittels Totalendoprothese (Hemiprothese: ATZ *n* = 2541; 83 % vs. ATZ/EPZ *n* = 3111; 79,1 %, Totalendoprothese: ATZ *n* = 520; 17 % vs. ATZ/EPZ *n* = 822; 20,9 %, *p* < 0,001).

Die Mortalität im Krankenhaus war in Kliniken mit dualer Zertifizierung (*n* = 257; 6,4 %) gegenüber Kliniken mit zertifiziertem ATZ (*n* = 158; 4,7 %) signifikant erhöht (*p* = 0,003).

In der multivariaten Analyse zeigten sich für zertifizierte ATZ-Kliniken eine erhöhte Wahrscheinlichkeit für eine gelenkerhaltende operative Versorgung (OR 3,61; *p* < 0,001) und für eine Entlassung nach Hause bzw. ins betreute Wohnen (OR 1,14; *p* = 0,023), aber eine geringere Wahrscheinlichkeit für eine stationäre Wiederaufnahme und eine Reoperation (Tab. 3). Es erfolgte nach der Entlassung aus dem ATZ/EPZ häufiger eine stationäre Weiterbehandlung, wobei, methodisch bedingt, nicht zwischen geriatrischer Fachklinik (GFK) oder einer sonstigen stationären Weiterbehandlung unterschieden werden kann.

**Tab. 3:** Akutphase: multivariable Analyse (logistische Regressionen für diskrete Variablen und lineare Regressionen für kontinuierliche Variablen. Adjustiert auf Alter, Geschlecht und ASA-Score).

Einfluss von EPZ + ATR-Kliniken auf … (Nur ATR-Kliniken als Referenz)	*N*		OR		95 %-KI vom OR	*p*-Wert	
**Akutphase**							
*Verstorben im Krankenhaus*							
Nein (Referenz) Ja	7339		1,26		[1,02; 1,56]	0,031	
*Mobilität nach 7 Tagen*							
Keine Gehfähigkeit vorhanden (Referenz) Selbstständig/mit Hilfsmittel	7103		0,91		[0,80; 1,02]	0,112	
*Reoperation in Akutphase*							
Nein (Ref.) Ja	7349		1,03		[0,81; 1,31]	0,839	
*Entlassen nach …*							
Nach Hause/betreutes Wohnen (Ref.) Nicht nach Hause	6665		1,14		[1,02; 1,28]	0,023	
*Versorgung/Operationstyp*							
Gelenkerhalt (Referenz) Endoprothetik	7359		3,63		[2,89; 4,60]	< 0,001	
–	***N***		**ẞ**		**95** **%-KI von ẞ**	***p*-Wert**	
Zeitspanne: Aufnahme bis Operation (h)*	7316		−2,16		[−3,49; −0,84]	0,001	
*Liegedauer im Krankenhaus (Tage)*	7310		−0,22		[−0,628; 0,184]	0,284	

In der ATZ/EPZ-Gruppe zeigten sich eine erhöhte Wahrscheinlichkeit für eine Wiederaufnahme in ein Krankenhaus (OR 1,45; *p* = 0,024), eine erhöhte Reoperationsrate (OR 1,42; *p* = 0,043) und eine signifikant erhöhte Mortalität (OR 1,26; *p* = 0,031) im Krankenhaus für die Akutphase, es bestand jedoch kein signifikanter Unterschied im 120-Tages-Intervall (Tab. 4).

**Tab. 4:** *120-Tage Follow-up*: multivariable Analyse. Logistische Regressionen. Adjustiert auf Alter, Geschlecht und ASA-Score

Einfluss von Endo + ATR-Kliniken auf … (Nur ATR-Kliniken als Referenz)	*N*	OR	95 %-KI vom OR	*p*-Wert
**120-Tage-Follow-up**				
*Verstorben zwischen Entlassung und 120-Tage-FU*				
Nein (Referenz) Ja	2827	0,87	[0,69; 1,09]	0,227
*Wiederaufnahme während 120-Tage-FU*				
Nein (Referenz) Ja	3301	1,45	[1,06; 2,02]	0,024
*Reoperation in 120-Tage-Follow-up*				
Nein (Ref.) ja	3129	1,42	[1,02; 2,00]	0,043

## Diskussion

Dislozierte Schenkelhalsfrakturen stellen im geriatrischen Patientenkollektiv eine interdisziplinäre Herausforderung dar, und ihre medizinische und gesundheitsökonomische Relevanz wird aufgrund der demografischen Entwicklung weiter zunehmen [13, 14].

Betroffene Patienten haben ein erhöhtes Letalitätsrisiko, zudem sind ihre Mobilität und Selbstständigkeit infolge der Verletzung stark gefährdet. Im Gegensatz zur elektiven Endoprothetik kann bei der Frakturendoprothetik eine präoperative Optimierung von Risikofaktoren und Komorbiditäten nur im begrenzten Umfang stattfinden [15]. Vor diesem Hintergrund sind die fachübergreifende Kooperation und das geriatrische Co-Management prognostisch von essenzieller Bedeutung [16].

Patienten über 75 Jahren haben im Vergleich zu den Patienten zwischen 65 und 74 Jahren ein erhöhtes Risiko, infolge einer Schenkelhalsfraktur zu versterben [17]. Je nach untersuchter Population sind 25–75 % der Patienten, die bislang Selbstversorger waren, ein Jahr nach der Fraktur nicht in der Lage, selbstständig zu gehen oder ihr altes Aktivitätsniveau zu erreichen [18].

Die Entscheidung zwischen Osteosynthese und Endoprothese bei Schenkelhalsfrakturen ist kontrovers diskutiert [19] und unterliegt regionalen und systemischen Unterschieden. Giordano et al. wiesen in einer rezenten Analyse nach, dass die Subspezialisierung der Operateure einen wesentlichen Einfluss auf die chirurgische Entscheidungsfindung, Eingriffsdauer und Entlassungsfähigkeit nach Hause hatte [20].

Wir konnten keinen signifikanten Unterschied hinsichtlich des Zeitfensters bis zur operativen Versorgung bei Kliniken mit einfacher bzw. dualer Zertifizierung nachweisen. Eine andere Studie zeigte eine signifikant längere Zeit bis zur operativen Versorgung in Level-I-Traumazentren mit 19,2 h im Vergleich zu Level-II/III-Traumazentren mit 16,8 h. Ein Zusammenhang zwischen dem Zeitfenster bis zur operativen Versorgung und der innerklinischen Mortalität konnte nicht nachgewiesen werden [21]. Die Überlebensrate zeigt sich insgesamt bei Patienten, die innerhalb von 24 h operativ versorgt werden, höher [22].

In den von uns ausgewerteten Daten des ATR-DGU zeigt sich, dass bei dislozierten Schenkelhalsfrakturen im geriatrischen Kollektiv die Mehrzahl der Fälle endoprothetisch versorgt wurde (*n* = 6999, 94,7 %); lediglich 390 Fälle wurden osteosynthetisch versorgt (5,3 %), diese Verteilung spiegelt die aktuelle Studienlage wider [23].

Die wesentliche Erkenntnis dieser Arbeit ist, dass bei geriatrischen Patienten mit dislozierten Schenkelhalsfrakturen signifikante Versorgungsunterschiede in Abhängigkeit vom Zertifizierungsstatus der behandelnden Klinik bestehen: In Kliniken mit einem zertifizierten AltersTraumaZentrum DGU® werden signifikant häufiger osteosynthetische Operationsverfahren gewählt (8,6 %), im Gegensatz zu Kliniken mit gleichzeitiger Zertifizierung als ATZ und EPZ, in denen deutlich seltener osteosynthetische Versorgungen favorisiert werden (2,6 %). Die Wahrscheinlichkeit für eine osteosynthetische Versorgung war auch in der multivariaten Analyse signifikant für ATZ erhöht (OR 3,63; 2,89–4,6; *p* < 0,001).

Eine Umfrage aus Ontario (Kanada) zeigte, dass neben patienten- und operateursbezogene Faktoren auch finanzielle und institutionelle Aspekte die Versorgungsrealität beeinflussen [24]. Den stärksten Effekt auf die Entscheidung einer Hemi- vs. Totalendoprothese bei Patienten mit Schenkelhalsfraktur > 60 Jahren hatten demnach das Patientenalter, der behandelnde Chirurg und die Klinik [25].

Die Implantation einer Totalendoprothese erfolgte im Vergleich zur Hemiprothese in Kliniken mit ATZ und EPZ signifikant häufiger. Hier könnten strukturelle Unterschiede der Kliniken und Erfahrung der Operateure bei der Wahl des Implantats eine Rolle spielen. Aufgrund des höheren Instabilitätsrisikos nach Totalendoprothesenimplantation sind eine gute Patienten-Compliance und ein konsequentes Nachbehandlungsschema erforderlich [26, 27].

Somit könnte nicht allein das Alter des Patienten, sondern der präoperative Zustand für die Wahl zwischen Totalendoprothese und Hemiprothese wichtig sein. Für gesündere und mobilere Patienten könnte die Totalendoprothese die bessere Wahl sein [28].

In der Literatur wird eine innerklinische Mortalität von 6 % beschrieben [29], diese ist vergleichbar mit der beobachteten Mortalität von 6,4 % für Kliniken mit ATZ und EPZ, wobei in ATZ-Kliniken ohne EPZ eine geringere Mortalität von 4,7 % beobachtet werden konnte. Die adjustierte multivariate Analyse bestätigte den Überlebensvorteil jedoch nur für die Akutphase, nicht mehr im Verlauf über 120 Tage.

Innerhalb von 120 Tagen zeigte sich jedoch eine signifikant erhöhte Rate an stationären Wiederaufnahmen und Reoperationen an Kliniken mit dualer Zertifizierung, und diese können als Surrogatparameter für erhöhte Behandlungskosten betrachtet werden.

Insgesamt finden sich in der Literatur nur wenige Angaben zu Reoperations- und Reaufnahmeraten. In Zentren, die das Follow-up nach 120 Tagen durchgeführt haben, wurden eine Reaufnahmerate von 10 % und eine Reoperationsrate von 6 % anlässlich der zuvor behandelten Fraktur erfasst [30]. Im Hinblick auf die Arten der Folgeoperationen zeigte sich ebenfalls ein variables Bild. An ATZ-Kliniken erfolgte häufiger eine Reoperation aufgrund von periprothetischen Frakturen; Entfernungen von Implantaten bzw. die Anlage einer Girdlestone-Situation wurden häufiger an Kliniken mit ATZ und EPZ indiziert. In jeweils 18 Fällen musste die Hüfte postoperativ erneut reponiert werden, wobei in Kliniken ohne EPZ weniger Endoprothesen eingesetzt wurden als möglicher Hinweis auf eine tendenziell erhöhte Rate an Instabilitäten. Nachweislich stellt ein geringes Fallvolumen der Klinik bzw. des Operateurs den entscheidenden Risikofaktor für Revisionen jeglicher Art dar [31].

Als wesentliche Limitation der Studie im Hinblick auf die Mortalität und Reoperationsrate ist die begrenzte Nachbeobachtung von 120 Tagen aufzuführen, da die Ergebnisse stark vom Zeitverlauf und vom Komplikationsprofil abhängig sind. In diesem Zusammenhang ist von besonderer Bedeutung, dass sich die Pseudoarthrose bzw. die avaskuläre Hüftkopfnekrose als wesentliche Komplikationen der Osteosynthese [32, 33] erst nach einem längeren Follow-up-Zeitraum zeigen würden, und somit in dieser Studie systematisch unterschätzt werden könnten.

Collinge et al. evaluierten gelenkerhaltende Versorgungen aus 26 US-amerikanischen Level-I-Traumazentren und identifizierten eine hohe Rate an technischen Fehlern (OR 5,29 bei mäßiger bis schlechter Reposition) und bei 21 % ein konsekutives Fixationsversagen bzw. eine Pseudarthrose [34].

Eine norwegische randomisierte kontrollierte Studie mit einem Nachbeobachtungszeitraum von 24 Monaten dokumentierte eine Reoperationsrate von 20 % für osteosynthetisch versorgte Schenkelhalsfrakturen und nur 5 % für hemiendoprothetisch versorgte Patienten > 70 Jahre, allerdings zeigte sich kein Vorteil im Harris Hip Score (HHS) als Maß für die Hüftgelenkfunktion [35]. Murphy et al. verglichen die Reoperationsrate innerhalb von 12 Monaten postoperativ und beobachteten signifikant mehr Reoperationen nach einer Osteosynthese bei dislozierter Schenkelhalsfraktur (38 % vs. 7 %) gegenüber Hemiendoprothesen [36].

Ramadanov et al. haben in einer Metaanalyse Risikofaktoren und Prädiktoren für das funktionelle Outcome nach Hüftgelenktotalendoprothesen untersucht und konnten das Patientenalter als Prädiktor für den HHS 6 Monate postoperativ und Schenkelhalsfrakturen als Prädiktor für die Komplikationsrate nachweisen [37].

Vor diesem Hintergrund postulieren einige Arbeiten eine Überlegenheit für die endoprothetische gegenüber der osteosynthetischen Versorgung im mittleren und im längerfristigen Verlauf [38], ohne jedoch einen Überlebensvorteil nachzuweisen. Eine Limitation dieser Arbeit stellt die Tatsache dar, dass wir keine Daten über zementiert bzw. zementfrei implantierte Hüftschäfte vorliegen haben. Einerseits entspricht bei einer Schenkelhalsfraktur (> 75 Jahren) die zementierte Schaftversorgung der aktuellen Studienlage [39, 40], andererseits wird v. a. bei schweren Vorerkrankungen eine hohe Prävalenz von fatalen Nebenwirkungen bei zementierten Versorgungen berichtet [41].

In diesem Kontext wird sich in Einzelfällen bei Gebrechlichkeit, Bettlägerigkeit und Altersdemenz entsprechend für die Osteosynthese entschieden [42].

Die konservative Therapie stellt bei proximalen Femurfrakturen die absolute Ausnahme dar. Auch bei nicht oder nur gering dislozierten Frakturen oder bettlägerigen Patienten zur Schmerzreduktion und besseren Pflege ist häufig die Indikation zur operativen Versorgung gegeben [43]. Die US-amerikanische Leitlinie *Management of Hip Fractures in Older Adults* sieht die Indikation zur konservativen Therapie höchstens bei nichtdislozierten Frakturen [44]; in anderen Leitlinien findet die konservative Therapie keine Erwähnung [45, 46].

Die dislozierte Schenkelhalsfraktur im geriatrischen Patientenkollektiv stellt außer Frage eine Hochrisikokonstellation, in der das operative Vorgehen bestmöglich mit den Betroffenen, Angehörigen und im interdisziplinären Konsens abgestimmt werden sollte, dar.

Vor diesem Hintergrund sind die Ergebnisse dieser Arbeit zur Versorgungsvarianz als auch zur Mortalität in der Akutphase und dem Kurzzeitverlauf spannend und diskussionswürdig. Weitere Untersuchungen sind erforderlich, um die Versorgungsvarianz und die unterschiedliche Rate an schwerwiegenden Komplikationen zu analysieren, um die Patientensicherheit und Ergebnisqualität zu verbessern.

## Zusammenfassung

In Deutschland liegt bei geriatrischen dislozierten Schenkelhalsfrakturen eine institutionelle Versorgungsvarianz vor. In alterstraumatologisch zertifizierten Kliniken ohne Endoprothetikzentrum zeigt sich eine signifikant erhöhte Rate an gelenkerhaltenden Versorgungen mit Unterschieden in Bezug auf die Morbidität und Mortalität in der Akutphase.

## Data Availability

Die Verfügbarkeit der Daten ist institutionell eingeschränkt. Die Daten stammen aus dem AltersTraumaRegister (ATR-DGU) und sind mit Genehmigung des Arbeitskreises AltersTraumaRegister (AK ATR) der Deutschen Gesellschaft für Unfallchirurgie (DGU) bei der Akademie für Unfallchirurgie (AUC) erhältlich.
